# Vitamin C Intervention for Critical COVID-19: A Pragmatic Review of the Current Level of Evidence

**DOI:** 10.3390/life11111166

**Published:** 2021-11-01

**Authors:** Patrick Holford, Anitra C. Carr, Masuma Zawari, Marcela P. Vizcaychipi

**Affiliations:** 1 Founder of Institute for Optimum Nutrition, Ambassador House, Richmond TW9 1SQ, UK; 2 Nutrition in Medicine Research Group, Department of Pathology & Biomedical Science, University of Otago, Christchurch 8140, New Zealand; anitra.carr@otago.ac.nz (A.C.C.); masuma.zawari@otago.ac.nz (M.Z.); 3 Faculty of Medicine, Imperial College, London SW7 2AZ, UK; m.vizcaychipi@imperial.ac.uk; 4 Intensive Care Medicine, Chelsea & Westminster Hospital, London SW10 9NH, UK

**Keywords:** vitamin C, SARS-CoV2, COVID-19, clinical trials, randomised controlled trials, intravenous vitamin C, pneumonia, sepsis, acute respiratory distress syndrome

## Abstract

Severe respiratory infections are characterized by elevated inflammation and generation of reactive oxygen species (ROS) which may lead to a decrease in antioxidants such as vitamin C and a higher requirement for the vitamin. Administration of intravenous vitamin C to patients with pneumonia and sepsis appears to decrease the severity of the disease and potentially improve survival rate. Severe acute respiratory syndrome coronavirus 2 (SARS-CoV-2) infection causes pneumonia, sepsis and acute respiratory distress syndrome (ARDS) in severe cases, and is referred to as coronavirus disease 2019 (COVID-19). Patients with COVID-19 infection also appear to have depleted vitamin C status and require additional supplementation of vitamin C during the acute phase of the disease. To date there have been 12 vitamin C and COVID-19 trials published, including five randomised controlled trials (RCTs) and seven retrospective cohort studies. The current level of evidence from the RCTs suggests that intravenous vitamin C intervention may improve oxygenation parameters, reduce inflammatory markers, decrease days in hospital and reduce mortality, particularly in the more severely ill patients. High doses of oral vitamin C supplementation may also improve the rate of recovery in less severe cases. No adverse events have been reported in published vitamin C clinical trials in COVID-19 patients. Upcoming findings from larger RCTs will provide additional evidence on vitamin supplementation in COVID-19 patients.

## 1. Introduction

Coronaviruses are single-stranded ribonucleic acid viruses comprising a lipid bilayer containing crown-like spikes (Latin, Corona = Crown) on their outer surface [1]. Infection with these viruses can affect both the upper and lower respiratory tract and can cause diseases ranging from a mild form, or common cold, to pneumonia [2]. In early December 2019, there were reports of infections with pneumonia-like symptoms of unidentified causes in China [3]. The infections were subsequently identified as being caused by a novel severe acute respiratory syndrome coronavirus 2 (SARS-CoV-2), the resultant disease being named coronavirus disease (COVID-19) in February 2020 [4]. Globally, as of August 2021, there have been over 200 million confirmed cases of COVID-19, including nearly 4.5 million deaths, reported [5]. Initial data indicated that the majority of patients were aged over 40 years, and that the risk of death increased with age [1]. Since then, numerous other risk factors have been identified, including specific underlying health conditions [6]. Currently, there is a global research effort to try and identify therapies that may help in the treatment of COVID-19.

Vitamin C is an essential nutrient that has important roles in immune function, including antioxidant, anti-inflammatory, antithrombotic, and immuno-modulatory functions [7,8]. Vitamin C deficiency, defined as a plasma concentrations of ≤11 µmol/L, is more common in the elderly, male gender, people with comorbidities, and low socioeconomic status [9]. These are also risk factors for COVID-19 infection [6]. Severe respiratory infections, such as pneumonia, are common clinical conditions that lead to a high requirement for vitamin C, thus providing grounds for active vitamin C replacement in patients who suffer from severe respiratory infections [10]. Although a vitamin C intake of 200 mg daily in healthy volunteers produces a saturating plasma concentration of 70 to 90 µmol/L [11,12], at least ten-fold higher doses (i.e., 2–3 g/day) are required to saturate the plasma of critically ill patients [13,14,15]. Critically ill patients are defined as patients at high risk for actual or potential life-threatening injuries and illnesses, requiring at least organ support and are continuously monitored in the intensive care unit (ICU). Vitamin C is generally administered parenterally to critically ill patients as it provides significantly higher circulating concentrations than enteral vitamin C [16].

Vitamin C administration in patients with pneumonia, sepsis and acute respiratory distress syndrome (ARDS) has shown potential benefits such as reducing duration of hospital and ICU stay and mortality [8]. Pneumonia, sepsis and ARDS are common complications of patients with severe COVID-19 and in March 2020, the World Health Organization highlighted vitamin C as a potential adjunctive therapy with biologic plausibility for patients with critical COVID-19 [17]. This pragmatic review summarizes the current level of evidence available from published studies (randomised controlled trials [RCTs] and retrospective cohort studies) that have investigated enteral and parenteral vitamin C supplementation in patients with SARS-CoV2 infection and severe COVID-19.

## 2. Vitamin C Status in Patients with COVID-19

Significant evidence indicates that patients with severe respiratory infections have depleted vitamin C status, with the prevalence of deficiency increasing with the severity of the condition [18,19,20]. The vitamin C status of patients with COVID-19 has been reported in several small observational studies (Table 1) [21,22,23,24,25,26]. Plasma concentrations of vitamin C in most of these patients were reported to be very low with 70–80% of the patients having hypovitaminosis C (plasma concentration <23 µmol/L) [22,24]. The low concentrations were despite patients receiving on average 124 mg/day vitamin C in their enteral or parenteral nutrition [26]. Interestingly, markers of oxidative stress were elevated in the COVID-19 patients relative to controls and there was an inverse correlation between oxidative stress markers and vitamin C status in the patients [25]. Thus, vitamin C supplementation appears warranted in these patients to address their hypovitaminosis C and restore adequate plasma vitamin C status [21]. It should be noted that short-term (i.e., 2–4 day) intervention with intravenous vitamin C may not be of sufficient duration to provide lasting benefit as 15–25% of patients can return to hypovitaminosis C status following cessation of intervention [15,27].

## 3. Randomised Controlled Trials with Intravenous Vitamin C

The first published randomised placebo-controlled trial was carried out in Wuhan, China, and administered IVC at a dose of 12 g/12 h at a late stage (10–17 days after the onset of the first symptoms) for seven days (Table 2) [28]. This trial reported a 70% reduced ICU and hospital mortality in patients with sequential organ failure assessment (SOFA) scores ≥3 who received IVC relative to those who received placebo (4 vs. 10 days, *p* = 0.03). There was no difference in invasive ventilation-free days of the intervention vs. placebo group overall (26.5 vs. 10.5 days, *p* = 0.56), however, this trial was halted early due to diminishing patient numbers. Nevertheless, increased peripheral capillary oxygen saturation/pulmonary function was observed in the IVC group relative to placebo (PaO_2_/FiO_2_; 229 vs. 151 mmHg, *p* = 0.01). Furthermore, the study group also had a lower inflammation marker (interleukin-6, IL-6) than the placebo group (19 vs. 158 pg/mL, *p* = 0.04). Patients with worse organ dysfunction may have more severe vitamin C deficiency [29], which could contribute to the benefit of intervention being more significant in the more severe COVID-19 patients with higher baseline SOFA scores in this study.

An open label RCT of 150 critical COVID-19 patients in Karachi, Pakistan, administered IVC at 50 mg/kg/day (3.5 g for 70 kg person) along with standard care or standard therapy alone and reported that the IVC group became symptom-free earlier (7.1 vs. 9.6 days, *p* < 0.0001), and spent fewer days in the hospital (8.1 vs. 10.7 days, *p* < 0.0001; Table 2) [30]. However, there were non-significant reductions in need for mechanical ventilation and mortality. A similar open label RCT in Tehran, Iran, randomised 60 patients with COVID-19 to 6 g/day IVC for five days or standard care [31]. Body temperature was reduced (*p* = 0.001) and oxygenation (SpO_2_) increased (*p* = 0.014) after three days of receiving the treatment. There were, however, no differences in ICU length of stay or mortality.

## 4. Retrospective Cohort Studies with Intravenous Vitamin C

A retrospective cohort study in Xi’an, Shaanxi, China included 76 patients with COVID-19 who received a loading dose of IVC 6 g/12 h on the first day, and 6 g once a day for the following four days compared to a cohort who received standard therapy alone (Table 3) [32]. A total of 48 (63%) patients had a diagnosis of moderate COVID-19, and 28 (37%) severe or critical disease. The median duration of symptoms before therapy was 12 days (8 to 16 days). Oxygen support status was improved compared with standard therapy (64% vs. 36%). Risk of 28-day mortality was reduced in the group who received IVC (HR = 0.14, 95% CI, 0.03–0.72, *p* = 0.037). Six (8%) patients with severe or critical disease died at the end of 28 days; one (17%) received IVC, and five (83%) standard therapy. The risks of mortality were even higher for patients who did not receive IVC if they had severe or critical disease (HR = 9.9, 95% CI, 1.8–54) or were aged >60 years (HR = 8.0, 95% CI, 1.2–51). Furthermore, C-reactive protein (CRP), procalcitonin (PCT) and interleukin-8 (IL-8) concentrations were reduced in the patients with COVID-19 who received IVC. Other retrospective cohort studies from Wuhan, China, also reported reductions in inflammatory markers (CRP, IL-6, TNF-α) and ameliorated cardiac injury in patients with severe COVID-19 who had received a loading dose of 100 mg/kg bodyweight 6 hourly on the first day followed by 100 mg/kg 12 hourly for the following 5 days, relative to those who received standard therapy only [33,34].

Severe COVID-19 is mainly characterized by deteriorating respiratory function and rapid progression of radiological lesions, while the critical type requires mechanical ventilation and is accompanied by shock or multiple organ failure. These two types are reported to be associated with a mortality rate as high as 66% [35]. To investigate the effect of high-dose IVC in the prevention of disease aggravation from moderate to severe, a study in Shanghai, China matched 110 patients with moderate COVID-19 pneumonia (55 per group) who received either IVC 100 mg/kg (7.5 g for 75 kg person) or standard care [35]. A third fewer patients progressed to severe status when given IVC (4 vs. 12; RR 0.28 [0.08, 0.93], *p* = 0.03). The duration (*p* < 0.001) and incidence (2/21 vs. 10/22, *p* = 0.08) of systemic inflammatory response syndrome (SIRS) and CRP levels (*p* = 0.05) were also reduced. Blood clotting time was improved (activated partial thromboplastin time, *p* = 0.02), and CD_4_^+^ (helper) T cell numbers were increased (*p* = 0.04).

Two retrospective cohort studies have reported no positive effects of IVC administration (Table 3). A retrospective study with propensity score matching in a New York ICU initiated IVC 1.5 g every six hours within 7 ± 5 days from admission (n = 8) for up to four days and compared the outcomes with untreated patients (n = 24) [37]. Patients in the IVC group had higher rates of hospital mortality (19 [79%] vs. 7 [88%], *p* = 0.049) and mean SOFA scores post-treatment (12 ± 3 vs. 8 ± 4, *p* < 0.005). There was no difference in the daily vasopressor requirement or in ICU length of stay between the treatment and comparator groups. However, it should be noted that the intervention group comprised only 8 patients and their mean SOFA score was nearly 3 points higher than the control group indicating greater severity of the treatment group at baseline. Furthermore, higher numbers of patients in the control group received convalescent plasma and prednisolone, which is highly suggestive that the standard of care may have been different between the patients included in this retrospective analysis. Another study in Ankara, Turkey administered IVC at 2 g/day initiated within a median duration of three days after admission (n = 153) compared with a group who received standard care alone (n = 170) [36]. There was a trend towards a decrease in duration of hospital stay (7 vs. 8 days, *p* = 0.05), no differences in re-admission rate (*p* = 0.94), admission to intensive care, need for advanced oxygen support (*p* = 0.49) or mortality (*p* = 0.52), but a higher need for advanced medical treatment (*p* < 0.001). However, as discussed below, emerging evidence is indicating that the vitamin C dosing regimens used in many of these studies may be inadequate in the critical stages of COVID-19.

## 5. Randomised Controlled Trials with Oral Vitamin C Supplementation

Fewer trials have investigated the effect of oral vitamin C in patients with COVID-19. A trial of 214 outpatients in the USA administered standard care, or vitamin C (8 g/day), or zinc (50 mg/day), or a combination of both vitamin C and zinc, and showed an 18% (1.2 day) reduction in the number of days to reach 50% reduction in symptoms in the participants who received the vitamin C (Table 4) [38]. Unfortunately, this trial was halted early resulting in a non-significant difference compared with the control group (*p* = 0.38). Nevertheless, independent statistical analysis of the results showed a 71% (*p* = 0.036) increase in the rate of recovery in the vitamin C group compared to standard care [39]. Thus, a better outcome might have been achieved if the trial had continued to the full sample size.

In another oral intervention study, hospitalised patients with non-severe COVID-19 in Isfahan, Iran, received low-dose oral vitamin C (1000 mg/day) plus oral vitamin E (400 IU/day) in addition to the national standard treatment regimen (hydroxychloroquine) or standard regimen alone [40]. There were no significant differences in the duration of hospitalisation (*p* = 0.82). A recent retrospective cohort study of patients who received low-dose oral vitamin C (1000 mg/day) indicated no association with hospital or 30-day mortality or organ injury, a longer ICU and hospital length of stay, but they did observe a significantly decreased incidence of thrombosis [41]. Overall, low-dose oral intervention appears to be less effective than high-dose oral and intravenous vitamin C.

## 6. Case-Control Study of Oral Vitamin C Supplementation

A hospital-based matched case-control study was conducted among health-care workers in India, from September to October 2020 [42]. Cases and controls were health-care workers who tested positive and negative, respectively, for SARS-CoV-2 infection. Out of 67 participants, 54 participants took a dose of 500 mg vitamin C once daily, and 13 participants took 500 mg twice daily. These participants were then matched with 305 participants who did not take any vitamin C. Vitamin C prophylaxis had no significant association with SARS-CoV-2 infection (OR 0.71, 95% CI, 0.40–1.26). The outcomes from this study correlate with previous research indicating that prophylactic vitamin C did not decrease the risk of the common cold in the general population, except in specific subgroups who experienced enhanced physical stress [43]. However, prophylactic vitamin C has been shown to decrease the risk of more severe respiratory conditions such as pneumonia [44]. As such, regular vitamin C supplementation may prevent mild SARS-CoV-2 infection from progressing to the more severe conditions of pneumonia and sepsis that are observed in critical COVID-19, thus potentially decreasing the need for hospitalisation and intensive care.

## 7. Safety of Oral and Intravenous Vitamin C

Uptake of oral vitamin C is regulated by the kinetics of the intestinal vitamin C transporter (SVCT-1), which limits the amount of vitamin C that can be absorbed at any one time to between 200 and 500 mg [11,45]. Thus, adverse events related to high-dose oral vitamin C are generally related to gastrointestinal disturbance from unabsorbed vitamin C passing through the intestines [46]. In contrast, intravenous vitamin C by-passes the regulated intestinal uptake of oral vitamin C, resulting in significantly higher circulating concentrations [16]. This elevated peak is still relatively transient, however, because vitamin C is water-soluble and is rapidly cleared by the kidneys, with a half-life of approx. 2–4 h depending on the administration regimen [47,48]. Nevertheless, circulating vitamin C concentrations can remain elevated if IVC is administered to people with renal dysfunction due to their attenuated ability to clear the infused doses.

Oxidative degradation of vitamin C can result in elevated oxalate concentrations which can potentially result in oxalate nephropathy, particularly in people predisposed to the condition [49,50]. However, because oxidised vitamin C (dehydroascorbic acid) is readily reduced back to vitamin C by both chemical and enzymatic means in the body, further degradation of dehydroascorbic acid to oxalate would only occur in the absence of other reducing agents. Furthermore, in the ICU setting, patients with renal insufficiency generally receive intermittent or continuous haemodialysis, which decreases circulating vitamin C concentrations [51]. People with glucose-6-phosphatedehydrogenase deficiency can experience haemolytic anaemia with high-dose IVC administration due to an inability to neutralise excess hydrogen peroxide, however, this is less likely to occur at the lower divided doses administered in ICU settings [52]. Spurious point-of-care glucometer readings can occur following IVC administration due to interference of high concentrations of vitamin C with specific glucometer biochemistry [53]. At the doses used in the ICU setting, this generally only occurs in patients with renal impairment [54].

To date, no adverse events have been reported in the published vitamin C and COVID-19 clinical trials (Table 2, Table 3 and Table 4). There have been two cases reported in the literature of oxalate nephropathy in patients with COVID-19 who were administered IVC at a dose of 50 mg/kg 4× daily, although the cause of the acute kidney injury was acknowledged as likely multifactorial [55]. A third case occurred in a kidney transplant recipient with COVID-19 who had apparently consumed oral vitamin C (500 mg 3× daily), however, the connection to vitamin C was extremely tenuous due to no evidence of elevated oxalate concentrations in their blood or urine [56]. Thus, there is no evidence that vitamin C is harmful when the above contraindications are taken into consideration [49].

## 8. Future Directions

There are currently numerous trials up and running around the world investigating vitamin C for the prevention and treatment of COVID-19. A number of these trials comprise combination therapies with other nutrients and/or medications, such as dexamethasone, however it will be difficult to tease out the contribution of vitamin C to any observed effects in these trials. Although pharmacokinetic studies have indicated that doses of vitamin C of 2–3 g/day can saturate the blood of critically ill patients [14,15], it is likely that higher doses will be required to optimise tissue levels due to possible downregulation of cellular vitamin C transporters as a result of elevated inflammation [57]. This premise is supported by trials which have shown dose-dependent effects of vitamin C administration [58]. Thus, future trials should explore optimisation of dosing regimens. Early administration of vitamin C is also likely to have greater benefit [59,60].

Two large monotherapy RCTs are currently underway internationally (LOVIT-COVID NCT04401150 and REMAP-CAP NCT02735707) with IVC dose and duration of 200 mg/kg/day for 4 days. Of note, these trials will be assessing survivor health-related quality of life at 6 months. This is relevant to the long-term complications of COVID-19, or what is being referred to as ‘long COVID’ [61,62]. However, as has been alluded to above, cessation of IVC after only a few days may result in return to hypovitaminosis C status in some patients, and is thus unlikely to have an impact on long term quality of life outcomes. Therefore, moving forward, trials may want to adopt a more pragmatic design whereby patients receive IVC whilst hospitalised and then move to oral vitamin C following discharge to determine if this has a greater impact on the ongoing complications of long COVID.

## 9. Conclusions

Observational studies have indicated that patients with COVID-19 have high rates of hypovitaminosis C and vitamin C deficiency, which are comparable to patients with sepsis and septic shock [7]. Previous studies of high-dose IVC administration in patients with sepsis and ARDS have shown reduced duration of ICU and hospital stay as well as decreased mortality [27,63]. Based on the evidence from preliminary RCTs and retrospective cohort studies, vitamin C appears to also support positive outcomes in COVID-19 in both inpatient and outpatient settings, leading to a beneficial effect in patients with moderate symptoms, as well as patients with pneumonia, sepsis and ARDS. Intervention with IVC resulted in improved oxygenation and pulmonary function, reduced inflammatory markers and temperature, decreased days in ICU and hospital, and decreased mortality, particularly in the more severely ill patients. Oral supplementation in mild to moderate cases also increased the rate of recovery when taken in high-doses.

Overall, the intervention studies to date have a number of limitations such as small sample sizes and early termination of some studies, differences in vitamin C dose and duration and lack of optimisation of these, lack of placebo controls, and no pre-and post-intervention plasma vitamin C concentrations. However, living meta-analysis of the studies as they are published is supporting the premise that high-dose IVC can improve the complications associated with COVID-19 and potentially decrease mortality [64]. Notably, there have been no adverse events reported in the published trials to date.

## Figures and Tables

**Table 1:** Vitamin C status in patients with COVID-19.

Population Location	Method	Findings	Reference
18 patients with ARDS ^1^ Barcelona, Spain.	Plasma HPLC-PDA ^2^	17 patients had <8 µM vitamin C 1 patient had 14 µM vitamin C	[23]
21 ICU ^3^ patients Thornton, Colorado, USA	Serum	Total cohort (n = 21) had 22 µM vitamin C (45% were deficient, 70% were hypovitaminosis C) Survivors (n = 11) had 29 µM vitamin C Non-Survivors (n = 10) had 15 µM vitamin C	[22]
31 hospitalised patients 51 healthy controlsShanghai, China	Plasma UHPLC-MS ^4^	6 patients (no IVC ^5^) had 11 µM vitamin C 25 patients given 100 mg/kg/day IVC had 76 µM 51 healthy controls had 52 µM vitamin C	[21]
50 symptomatic patients 21 healthy controls Jigwa, Nigeria	Serum Colourimetric	Patients had 19 µM vitamin C Controls had 25 µM vitamin C	[25]
9 ICU patients with severe pneumonia Liège, Belgium		Patients had 22 µM vitamin C (reference range: 35–86 µM)	[26]
67 patients with ARDS Barcelona, Spain	Plasma HPLC	Mean vitamin C concentration was 8 ± 3 µM 55 patients (82%) had values <23 µM 12 patients (18%) had values <6 µM	[24]

^1^ ARDS: acute respiratory distress syndrome, ^2^ PDA: photo diode array, ^3^ ICU: intensive care unit, ^4^ UHPLC-MS: ultra-high-performance liquid chromatography-mass spectrometry, ^5^ IVC: intravenous vitamin C. Note: vitamin C concentrations <11 µM are considered deficient, and <23 µM are considered hypovitaminosis C.

**Table 2:** Randomised controlled trials investigating the effect of intravenous vitamin C (IVC) in patients with COVID-19.

Population Mean Age Location	Intervention Duration	Findings (IVC vs. Control)	Reference
54 patients with COVID-19-pneumonia and multiple organ injury Age = 67 ± 13 years Wuhan, Hubei, China	IVC ^1^ 24 g/day (n = 27) or placebo (n= 29) for 7 days	Higher PaO_2_/FiO_2_^2^ (229 vs. 151 mmHg, *p* = 0.01) Lower Interleukin-6 (19 vs. 158 pg/mL, *p* = 0.04) Lower ICU ^3^ and hospital mortality in patients with SOFA ^4^ scores ≥3 (4 vs. 10 days, *p* = 0.03) No difference in ventilation-free days (26.5 vs. 10.5 days, *p* = 0.56)	[28]
150 patients with severe COVID-19 Age = 52–53 years Karachi, Pakistan	IVC 50 mg/kg/day + standard therapy or standard therapy (75 per group)	Patients became symptom-free earlier (7.1 ± 1.8 vs. 9.6 ± 2.1 days, *p* < 0.0001) Patients spent fewer days in the hospital (8.1 ± 1.8 vs. 10.7 ± 2.2 days, *p* < 0.0001) No difference in need for mechanical ventilation (16% vs. 20%, *p* = 0.4) No difference in mortality (9.3% vs. 14.6%, *p* = 0.3)	[30]
60 patients with COVID-19 Age = 57–61 years Tehran, Iran	IVC 6 g/day + standard therapy or standard therapy (30 per group) for 5 days	Lower body temperature on 3rd day of hospitalisation (*p* = 0.001) Improvement in oxygen saturation on 3rd day of hospitalisation (*p* = 0.014) No differences in length of ICU stay or mortality	[31]

^1^ IVC: intravenous vitamin C, ^2^ PaO_2_/FiO_2_: ratio of partial pressure of oxygen to fraction of inspired oxygen, ^3^ ICU: intensive care unit, ^4^ SOFA: sequential organ failure assessment.

**Table 3:** Retrospective cohort studies investigating the effect of intravenous vitamin C (IVC) in patients with COVID-19.

Population Mean Age Location	Intervention Duration	Findings (IVC vs. Control)	Reference
76 patients with moderate to severe COVID-19 Age = 61 (52–71) years Xi’an, Shaanxi, China	IVC ^1^ 6 g/12 h on first day, 6 g/day for following 4 days + standard therapy (n = 30) or standard therapy (n = 46) for 5 days	Improved oxygen support status (64% vs. 36%) Reduced risk of 28-day mortality (HR ^2^ = 0.14, 95% CI, 0.03–0.72, *p* = 0.037) Reduced CRP ^3^, PCT ^4^ and IL-8 ^5^ concentrations	[32]
110 patients with moderate COVID-19 pneumonia Age = 36 (31–47) years Shanghai, China	IVC 100 mg/kg/day + standard therapy or standard therapy (55 per group) for 7 days	Fewer patients progressing to severe type (4 vs. 12; RR ^6^ 0.28 [0.08, 0.93], *p* = 0.03) Reduction in duration (*p* < 0.001) and incidence of SIRS ^7^ (*p* = 0.008) Lower CRP concentrations (*p* = 0.005) Lower activated partial thromboplastintime (*p* = 0.02) Higher CD_4_^+^ (helper) T cells (*p* = 0.0004)	[35]
232 patients with COVID-19-pneumonia Age = 60 ± 14 years Ankara, Turkey	IVC 2 g/day + standard therapy (n = 153) or standard therapy (n = 170)	Shorter length of hospital stay (7 vs. 8 d, *p* = 0.05) No difference in re-admission rate (*p* = 0.94), admission to ICU ^8^, need for advanced oxygen support (*p* = 0.49), and mortality (*p* = 0.52) Need for advanced medical treatment (*p* < 0.001)	[36]
34 critically ill patients with COVID-19 Age = 65 ± 12 years New York, USA	IVC 1.5 g/6 h + standard therapy (n = 8) or standard therapy (n = 24) for 4 days	Higher rate of hospital mortality (19 [79%] vs. 7 [88%], *p* = 0.049) and SOFA scores (12 ± 3 vs. 8 ± 4, *p* < 0.005) No difference in daily vasopressor requirement or ICU length of stay	[37]
236 patients with severe COVID-19 Age = 66 (57–73) years Wuhan, China	IVC 100 mg/kg/6 h on day 1 then 100 mg/kg/12 h for the next 5 days + standard therapy (n = 85) or standard therapy (n = 151)	Reduction in inflammatory markers (CRP, *p* = 0.032; IL-6, *p* = 0.005; TNF-α ^9^, *p* = 0.015)	[33]
113 patients with severe COVID-19 and cardiac injury Age = 68 (59–77) years Wuhan, China	IVC 100 mg/kg/6 h on day 1 then 100 mg/kg/12 h for the next 5 days + standard therapy (n = 51) or standard therapy (n = 62)	IVC was associated with ameliorated cardiac injury (OR 2.42 [1.02, 5.73], *p* = 0.04) Reduced levels of inflammatory markers (CRP, IL-6, IL-8, TNF-α) at 21 days of hospitalisation	[34]

^1^ IVC: intravenous vitamin C, ^2^ HR: hazard ratio, ^3^ CRP: C-reactive protein, ^4^ PCT: procalcitonin, ^5^ IL: interleukin, ^6^ RR: relative risk, ^7^ SIRS: systemic inflammatory response syndrome, ^8^ ICU: Intensive care unit, ^9^ TNF: tumor necrosis factor.

**Table 4:** Studies investigating the effect of oral vitamin C in patients with COVID-19.

Population Mean Age Location	Intervention Duration	Findings (Vitamin C vs. Control)	Reference
**Randomised Controlled Trials**			
214 patients with SARS-CoV-2 Age = 45 ± 15 years Ohio and Florida, USA	8 g/day oral vitamin C or 50 mg/day zinc gluconate or vitamin C + zinc gluconate or standard care (n = 48–58 per group) for 10 days	18% (1.2 day) decrease in time to 50% reduction of symptoms (*p* = 0.38) Vitamin C increased the rate of recovery by 71% (*p* = 0.036)	[38]
			[39]
72 non-serious hospitalised patients Mean age = 36 years Isfahan, Iran	1000 mg/day oral vitamin C (plus 400 IU/day vitamin E) or standard care (n = 34–38 per group) until hospital discharge or ICU admission	No differences in clinical improvement or duration of hospitalisation (*p* = 0.82) No patients died in the study	[40]
**Retrospective cohort study**			
296 critically ill patients Age = 61 ± 15 Riyadh, Saudi Arabia	1000 mg/day oral vitamin C or standard care (n = 148 per group) for approx. 11 days	No association with hospital or 30-day mortality, or organ injury Longer ICU and hospital length of stay Decreased incidence of thrombosis (6 vs. 13%, OR 0.42 [0.18–0.94], *p* = 0.03)	[41]

## Data Availability

Not applicable.
